# ‘How Did That Make You Feel?’ Latinas' Use of Genetic Counseling and Testing for Hereditary Cancer Risk After Watching a Culturally Targeted Video and Receiving Patient Navigation

**DOI:** 10.1002/pon.70261

**Published:** 2025-08-29

**Authors:** Sara Gómez‐Trillos, Pilar Carrera, Amparo Caballero, Vanessa B. Sheppard, Kristi D. Graves, Beth N. Peshkin, Marc D. Schwartz, Claudia Campos, Nathaly Garcés, Alejandra Hurtado de Mendoza

**Affiliations:** ^1^ Lombardi Comprehensive Cancer Center Georgetown University Medical Center Washington District of Columbia USA; ^2^ Fisher Center for Hereditary Cancer and Clinical Genomics Research Washington District of Columbia USA; ^3^ Grupo de Investigación Psicología Salud y Sociedad Universidad CES Medellín Colombia; ^4^ Social Psychology and Methodology Department Universidad Autónoma de Madrid Madrid Spain; ^5^ School of Population Health Virginia Commonwealth University Richmond Virginia USA; ^6^ Nueva Vida, Inc Alexandria Virginia USA; ^7^ Ralph Lauren Center for Cancer Prevention Washington District of Columbia USA

**Keywords:** cancer, emotions, genetic counseling, health communication, health education, hereditary breast or ovarian cancer syndrome, Hispanic or Latino, oncology

## Abstract

**Objective:**

Culturally targeted narrative education is a promising approach to cancer prevention and control. This study evaluates the uptake of genetic counseling and testing (GCT) in Latinas at risk for hereditary breast and ovarian cancers (HBOC) after watching a culturally targeted narrative video and being navigated to GCT services.

**Methods:**

Latina women at increased risk for HBOC were recruited through community‐based organizations. Participants responded to surveys before and after watching Spanish‐language telenovela‐style video. Surveys measured sociodemographic and clinical variables, HBOC and GCT knowledge, transportation with the story, identification with characters, and emotions elicited by the video. After watching video, participants were offered patient navigation services to free or low‐cost GCT and completed a 3‐month follow‐up phone survey to assess GCT uptake.

**Results:**

Participants (*N* = 40) were 47.35 years old on average (SD = 9.48); all were born outside the United States. At the 3‐month follow‐up (*N* = 37), 27 (72.9%) and 26 (70.27%) participants had attended genetic counseling and genetic testing, respectively. U Mann Whitney tests found statistically significant differences between women who attended counseling versus those who did not at baseline knowledge (*U* = 216.00, *p =* 0.000) and distress elicited by the video (*U* = 73.5, *p =* 0.03). A logistic regression with *distress elicited by the video* as a predictive variable reached statististical significance (*β* = −0.27, *p* = 0.037, CI 95% 0.58–0.98).

**Conclusions:**

GCT uptake was promising, supporting a role for culturally targeted narrative video education along with a patient navigation component in increasing interest in cancer prevention and reducing healthcare disparities in HBOC genetic services.

**Trial Registration:**

NCT03075540 (Initial release 2/22/2017)

## 1 Introduction

Despite significant advances in cancer genomics, the translation of this knowledge into practice has lagged for racially and ethnically minoritized populations [1]. People who carry a known pathogenic variant (PV) have an increased risk of developing certain cancers during their lifetime [2]. In the context of hereditary breast and ovarian cancer (HBOC) risk, a PV in the *BRCA1* or *BRCA2* (*BRCA1/2*) genes increases the risk of developing breast and ovarian cancers by up to 87% and 63% by age 70 [3]. Knowing a person's *BRCA1/2* status is clinically actionable for the person tested and medically relevant for their first‐ and second‐degree relatives [3]. For example, a positive *BRCA1*/2 result may inform decisions about risk‐reducing surgeries, earlier and more frequent screening, and cascade testing for family members. Receiving genetic counseling prior to testing is highly recommended as genetic counseling increases knowledge, satisfaction with medical decisions, and decreases anxiety [4, 5]. For decades, Black and Hispanic/Latinx women have been less likely to be referred to and receive genetic counseling and testing compared to their non‐Hispanic White peers, further exacerbating disparities in cancer outcomes [6, 7, 8].

A recent systematic review reveals that awareness remains among the most commonly cited barriers that Hispanic/Latinx women face to accessing genetic counseling and that it is particularly low among Spanish‐speakers [9]. Not surprisingly, the few interventions that have targeted a Hispanic/Latinx population in cancer genomics have been primarily educational, including pre‐recorded slide deck presentations with audio, web‐based videos delivered by avatars, and educational booklets [10, 11, 12, 13]. While all of these strategies were successful in their intended outcomes (e.g., to improve patient preparedness for pre‐test counseling [11] and increase knowledge [10]) only one has been found to enhance genetic counseling uptake in this population [12]. Given the potential of genetic counseling and testing (GCT) to provide life‐saving health information, increasing uptake of GCT use is paramount.

Culturally targeted narrative education is a promising way forward in cancer prevention and control [14]. Culturally targetting health information is more effective for minoritized populations as it enhances health communication at a cultural, linguistic, and literacy level, making it more personally relevant [11]. Narratives can address the informational needs and preferences of individuals from backgrounds in which oral tradition is prevalent, those with lower health literacy and low English proficiency, making them an ideal fit for Hispanic/Latinx people, the fastest growing ethnic group in the United States [15, 16, 17].

Results of culturally targeted interventions using video presentations with narrative components have revealed that Latina participants highly enjoy and endorse the story format [10, 11]. Presenting health information in a narrative (vs. factual) form has been shown to improve attention and recall and enhance emotional responses [16, 18, 19]. Additionally, in the context of cancer care, when information may be especially hard to process, narratives can improve information processing and reduce both counterarguments and resistance [16]. Narratives that are culturally targeted further reinforce personal relevance, enabling feeling transported with the story (i.e., transportation) and identification with the characters [20, 21]. Thus, culturally targeted narratives can be particularly powerful motivators for behavior change.

Video interventions in particular are an appealing educational approach to engage historically minoritized communities in reserch [22, 23]. Video interventions that have used both narrative and culturally tailored video approaches in cancer education have had encouraging results. For instance, *“Tamale Lesson,”* a narrative video providing cervical cancer education, increased screening for cervical cancer in Mexican‐American women [24]. In another study, Henderson et al. [25] developed and piloted a narrative decision‐aid video for Black women at increased risk for HBOC. Participants reported high satisfaction with the video and increased intentions to attend genetic counseling after watching the video [25].

Our team developed a culturally targeted narrative video in a telenovela style to enhance the uptake of genetic counseling for Latina women at increased risk for HBOC. The video incorporates key constructs from the theory of planned behavior, health communication, the literature on the influence on emotions in behavioral change, and our extensive formative research [26, 27, 28]. The findings from the formative research and the constructs that influenced the development of the video have been reported in detail previously [29, 30]. The goals of this single arm trial were to: (1) assess the uptake of genetic counseling and testing in Latinas at risk for HBOC within 3‐months of watching our culturally targeted video and being navigated to GCT services (2) explore which factors are associated with uptake of genetic counseling uptake at 3 months.

## 2 Methods

### 2.1 Participants

Women were eligible if they self‐identified as a Hispanic/Latina, were 21 years old or older, were fluent in Spanish, had never received genetic counseling or testing, and met the National Comprehensive Cancer Network (NCCN) criteria for hereditary breast and ovarian genetic cancer risk assessment at the time of the study.

### 2.2 Study Procedures

#### 2.2.1 Recruitment

We partnered with two‐community based organizations (CBOs) serving the Hispanic/Latinx population in the Washington, DC metropolitan area. Both CBOs provide breast care services and patient navigation in Spanish. Patient navigators at the CBOs identified potential participants for the study and referred them to a trained and bilingual study Research Assistant (RA) to confirm eligibility. We shared study flyers in public places, including hospitals, and with stakeholders. The RA spoke with potential participants over the phone to screen them, confirm eligibility, and assess interest in participating in the study. Data were collected until September 2019.

**FIGURE 1:**
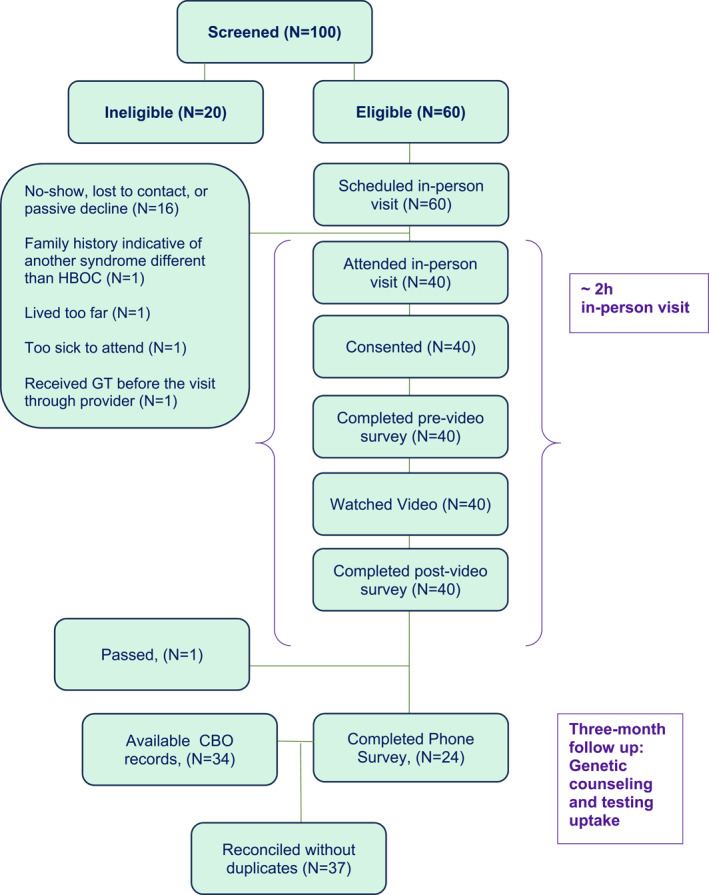
CONSORT diagram.

Eligible and interested participants were scheduled for an in‐person visit at either the CBO or the RA's office. Reimbursement for transportation cost was provided when needed. Figure 1 shows the CONSORT diagram.

#### 2.2.2 In‐Person Visit

The in‐person visit, which lasted between 1.5 and 2 h, took place in a private office with only the RA and participant present. Trained RAs explained the study in detail in lay‐language and obtained verbal informed consent. A copy of the informed consent form was shared with all participants for their own records.

Participants were asked to respond to a 25‐min survey verbally (i.e., RAs asked the questions out loud and documented answers). Immediately after completing the baseline survey, all participants watched *“Is my cancer hereditary? Rosa Visits a Genetic Counselor*” 18‐min video. Then, participants responded to a post‐video 25‐min survey following the same procedures as before. Lastly, they responded to a short open‐ended interview about the video. Interviews were audio recorded with participants' permission and were 16 min long on average. The results from short interviews are reported elsewhere [29].

#### 2.2.3 Referral to Genetic Counseling and Testing

At the end of the in‐person visit, the RA asked participants whether they wished to be referred to free genetic counseling for hereditary cancer risk. If participants indicated interest, the RA informed the patient navigator at the appropriate CBO. Patient navigators were bilingual Latina women. Most were familiar with study participants as they were recruited through the two partner CBOs and already received navigation and support from the CBO for their care.

Patient navigators referred and navigated interested women to free or low cost genetic counseling, acting as a bridge between the research team, the patient, and the counselor. Navigators obtained additional consent from patients to initiate the referral process, completed the required forms, and referred them to free genetic counseling either at a local hospital or a pro‐bono private clinic that offered in‐person and virtual care. Navigation included either referring partcipants to a nurse coordinator at a hospital that took over the patient care or performing the different navigation tasks, including scheduling the appointment, sending reminders, attending GC appointments if needed, and checking in at the end of the GC to gauge understanding. Notably, our partner CBOs had already established these processes and collaborations outside of this research study, and the clinical and follow‐up care followed each CBOs' usual practice. No additional details about the navigators contact time and activities was collected.

If two members of the same family participated in the study and wished to receive genetic counseling, they were offered pre‐test counseling together in the same session, and the testing recommendation was at the counselor's discretion.

#### 2.2.4 Phone Survey

Three months after the in‐person visit, participants responded to a 5‐min survey via phone to assess genetic counseling and testing uptake.

All participants were compensated $30 for their time. Members of the same family were allowed to participate in the study and complete all study procedures simultaneously but in separate private offices with different RAs. All participants were given the option to take a break at any point in the in‐person visit if needed. Study procedures were approved by the Principal Investigator's main Institutional IRB. All study procedures were conducted in Spanish.

### 2.3 Video Description: “Is my Cancer Hereditary? Rosa Visits a Genetic Counselor”

As previously mentioned, the research team created an 18‐min Spanish‐language culturally targeted telenovela‐style video. Findings from the formative research, development, and pre‐post differences in psychosocial outcomes are presented elsewhere [27, 28, 29, 30, 31, 32]. In short, the video follows the story of Rosa, a Latina breast cancer survivor, who learns she is at risk for hereditary breast and ovarian cancer. The scenes portray Rosa's discussions with a doctor, family, and friends regarding GCT and model a genetic counseling session with an interpreter.

### 2.4 Measures

#### 2.4.1 Sociodemographic and Psychosocial Factors

##### 2.4.1.1 Sociodemographic and Clinical Factors

Age, ethnicity, race, country of origin, years living in the United States, marital status, highest education attained, and cancer status were assessed by self‐report and collected at baseline.

##### 2.4.1.2 Health Literacy

Health literacy was assessed using one item at baseline, “*How comfortable do you feel filling out medical forms by yourself?”* [15] Participants were considered as having low health literacy if they responded “*Not at all/Kind of…comfortable*” and high health literacy if they responded “*Pretty/Really/Extremely…confident*” to the question.

##### 2.4.1.3 Knowledge of HBOC and Genetic Counseling Risk Assessment (GCRA)

We captured knowledge about HBOC and GCT using two distinct scales at the pre and post‐video surveys. Knowledge about genetic counseling was captured with a validated 9‐item scale (Moffit Scale) [33]. Additionally, our team developed a 20‐item scale that tapped into the video's main messages regarding HBOC risk (GU‐Scale) [31].

Both scales had three response options: *True*, *False*, and *I don't know.* Participants were asked to choose one answer for each statement.

#### 2.4.2 Reactions to the Video

##### 2.4.2.1 Transportation

In narrative education, transportation refers to how engaged the audience is with the story. Using a 9‐item Likert type scale, we assessed transportation at the post‐video survey immediately after the participants had watched the video. The scale ranged from 1 = *not at all to* 7 = *very much* (e.g., *“I could picture myself in the scene of the events described in the narrative”*) [21], where higher scores represent higher levels of transportation. As the Cronbach's alpha was low (alpha = 0.5), we excluded the reverse coded items and one item with low loading leaving 6‐items in total (alpha = 0.7).

##### 2.4.2.2 Identification With the Main Character

At the post‐video survey we captured four components of identification with the main character, Rosa: liking (e.g., *How much do you like Rosa?*), similarity (e.g., *How similar do you think you are to Rosa?*), parasocial identification (e.g., *How much do you think you know Rosa?*), and wishful identification (e.g., *How much do you wish you were like Rosa?*) on a Likert type scale ranging from 1 = *Not at all* to 10 = *Very much* [34]. The average of these four items were used (alpha = 0.74). Higher scores mean higher levels of identification with Rosa.

##### 2.4.2.3 Emotions Elicited by the Video

6‐items on a Likert‐type scale ranging from 1 = *Not at all* to 10 = *Very much* were used to capture discrete emotions that the video elicited in participants at the post‐video survey. The scale was adapted from Murphy et al., 2013 [34] and informed by Yoo et al., 2014 [18]. The final scale included three negative emotions (e.g., “The video made me feel… *distress*/*sadness/fear*”) (alpha = 0.84) and three positive emotions (e.g., “The video made me feel… *happiness/hope/calm*”) (alpha = 0.64). Each emotions is treated as an independent variable.

##### 2.4.2.4 Interest in Genetic Counseling

At the end of the in‐person visit, participants were asked whether they wished to be referred to genetic counseling services *(yes/no).* Their answers were recorded by the RA.

#### 2.4.3 Primary Outcome

##### 2.4.3.1 Uptake of Genetic Counseling

Genetic counseling uptake was assessed by self‐report at the 3‐month follow‐up call. The research team confirmed referrals, attendance to genetic counseling, and testing completion with the patient navigators at each CBO. If any discrepancies occurred, the most recently available data was used. For participants who were lost to follow‐up at the 3‐month follow‐up, CBO records were used. If CBO records were not available, self‐reported measures were used.

#### 2.4.4 Secondary Outcome

##### 2.4.4.1 Uptake of Genetic Testing

Genetic testing uptake was assessed by self‐report at the 3‐month follow‐up call. The research team confirmed referrals, attendance to genetic counseling, and testing completion with the patient navigators at each CBO. If any discrepancies occurred, the most recently available data was used. For participants who were lost to follow‐up at the 3‐month follow‐up, CBO records were used. If CBO records were not available, self‐reported measures were used.

### 2.5 Data Analysis

#### 2.5.1 Descriptive Analysis

We calculated descriptive statistics to characterize the sample's sociodemographic and clinical factors. We report the frequency of participants who attended pre‐test genetic counseling and testing at the 3‐month follow‐up.

#### 2.5.2 Bivariate Analysis

To gauge which factors were associated with uptake to genetic counseling, we conducted exploratory analyses to assess differences in psychosocial variables (i.e., education, income, health literacy, cancer diagnosis, and knowledge) and reactions to the video (i.e., transportation, identification with the main character, and emotions elicited by the video) in women who attended versus did not attend a genetic counseling session.

We used chi‐square tests for dichotomous variables and, given the small sample size, Mann Whitney U tests for continuous and ordinal variables.

#### 2.5.3 Logistic Regression

Given the dichotomous dependent variable, we conducted an exploratory logistic regression to assess whether variables that were statistically significantly different between groups predicted genetic counseling participation. All statistical analyses were conducted in SPSS 29.

## 3 Results

### 3.1 Descriptive Analysis

#### 3.1.1 Sociodemographic and Clinical Factors

Sociodemographic and clinical factors are presented in Table 1. Participants (*N* = 40) were 47.35 years old on average (SD = 9.48), all were born outside of the United States, with 52.5% originally from El Salvador, followed by Perú (15%), Mexico (10%), and Guatemala (10%). Participants had been in the United States for an average of 16.19 years (SD = 9.25). Most participants had a high school education or lower (62.5%) and a household income below 40,000 USD a year (65%). 77.5% of participants had no health insurance at the time of enrollment. While all participants met NCCN criteria for HBOC, only 12 (30%) reported a personal history of cancer. None had received genetic counseling or testing for HBOC. Three pairs of women who participated were blood relatives (one pair were sisters, and two were a mother‐daughter dyad). Except for one participant, all others (*N* = 39; 97.5%) were interested in being referred to genetic counseling services after responding to the surveys and watching the video.

**TABLE 1:** Sociodemographic and clinical factors.

	*M* (SD)/*N* (%)
Age	47.35 (9.48)
Hispanic/Latina	40 (100%)
Race	
Black	0 (0%)
White	8 (20%)
More than one race	6 (15%)
Unknown	6 (15%)
Other	20 (50%)
Country of origin	
Bolivia	3 (7.5%)
Chile	1 (2.5%)
El Salvador	21 (52.5%)
Guatemala	4 (10%)
Mexico	4 (10%)
Peru	6 (15%)
Venezuela	1 (2.5%)
Years living in the U.S	16.19 (9.25)
Current marital status	
Divorced	5 (12.5%)
Married or living as married	24 (60%)
Never married	9 (22.5%)
Widowed	1 (2.5%)
Other	1 (2.5%)
Highest education attained	
High school or less	25 (62.5%)
Some college or more	15 (37.5%)
Annual household income	
< $40,000	26 (65%)
$40,000	10 (25%)
Prefer not to answer/missing	4 (10%)
Health insurance status	
Yes	9 (22.5%)
No	31 (77.5%)
Cancer diagnosis	
Breast cancer	10 (25%)
Ovarian cancer	2 (5%)
No personal history of cancer	28 (70%)
Health literacy	
Low	16 (40%)
High	24 (60%)

#### 3.1.2 Genetic Counseling and Testing Uptake

The genetic counseling uptake variable had three missing responses, one of which was from the participant who refused a GCT referral by the patient navigators. These three participants were excluded from the analysis, leaving a final sample of 37 participants (see Figure 1). 3 months after completing the in‐person visit, 27 (72.9%) participants had attended genetic counseling and 25 (92.59%) of those who attended genetic counseling completed genetic testing for hereditary cancer risk. One participant who did not receive genetic counseling completed genetic testing through her provider, so a total of 26 participants (70.27%) had received genetic testing at the 3‐month follow up (see Table 2).

**TABLE 2:** Genetic counseling and testing uptake at 3‐month follow‐up.

	Yes (*N*, %)	No (*N*, %)	Missing (*N*)	Total *N*
Interested in genetic counseling referral, (*N* = 40)	39 (97.5%)	1 (2.5)		40
Genetic counseling uptake, (*N* = 40)	27 (72.9%)	10 (27.03%)	3	37
Genetic testing uptake, (*N* = 40)	26 (72.22%)	10 (25%)	4	36

### 3.2 Bivariate Analysis

#### 3.2.1 Sociodemographic and Psychosocial Factors

Findings showed no statistically significant differences between those who attended genetic counseling compared to those who did not on variables of educational level (*U* = 105.00, *p =* 0.32), income (*U* = 133.5, *p =* 0.90), low versus high health literacy (*χ* ^2^ = 0.35, *p =* 0.55), cancer diagnosis (yes/no) (*χ* ^2^ = 2.69, *p =* 0.10), baseline knowledge measured by the Moffit Scale (*U* = 180.5 *p =* 0.07), and change in knowledge in both the Moffit (*U* = 110.5, *p =* 0.49) and GU Scales (*U* = 65.00, *p =* 0.18).

U‐Mann Whiney tests showed a statistical difference in baseline knowledge between those who attended counseling (*M* = 11.74, SD = 2.15) versus those who did not attend (*M* = 8.44, SD = 1.81) (*U* = 216.00, *p* = 0.000) measured by the GU‐Scale (see Table 3).

**TABLE 3:** Differences in women who attended versus did not attend genetic counseling.

Sociodemographic factors	Attended GC*N*, (%)Mean (SD)	No GC uptake*N*, (%)Mean (SD)	UMann‐whitney	Chi‐square	*p*‐value
Education level	11.85 (4.19)	13.10 (4.09)	*U* = 105.00		*p* = 0.32
	*N* = 27	*N* = 10			
Income	12.12 (23.94)	4.80 (3.23)	*U* = 133.5		*p* = 0.90
	*N* = 26	*N* = 10			
Low health literacy	11 (40.7%)	3 (30%)		*χ* ^2^ = 0.35	*p* = 0.55
	*N* = 27	*N* = 10			
Cancer diagnosis (yes/no)	6 (22.2%)	5 (50%)		*χ* ^2^ = 2.69	*p* = 0.10
	*N* = 27	*N* = 10			
Baseline knowledge Moffit scale	4.26 (1.71)	3.10 (1.45)	*U* = 180.5		*p* = 0.07
	*N* = 26	*N* = 10			
Baseline knowledge GU‐ scale	11.74 (2.15)	8.44 (1.81)	*U* = 216.00		*p* = 0.000**
	*N* = 27	*N* = 9			
Change in knowledge Moffit scale	0.92 (1.57)	1.30 (1.15)	*U* = 110.5		*p* = 0.49
	*N* = 26	*N* = 10			
Change in knowledge GU scale	3.83 (2.51)	5.37 (2.38)	*U* = 65.00		*p* = 0.18
	*N* = 24	*N* = 8			
Reactions to the video					
Transportation	4.93 (0.98)	4.98 (0.69)	*U* = 139.5		*p* = 0.74
	*N* = 26	*N* = 10			
Identification with the main character (four items)	8.23 (1.66)	8.25 (2.11)	*U* = 124.5		*p* = 0.72
	*N* = 27	*N* = 10			
Emotions elicited by the video					
Distress	2.26 (2.34)	4.60 (3.53)	*U* = 73.5		*p* = 0.03*
	*N* = 27	*N* = 10			
Sadness	4.04 (3.39)	4.20 (3.61)	*U* = 129.5		*p* = 0.85
	*N* = 27	*N* = 10			
Fear	3.11 (3.19)	3.10 (2.76)	*U* = 122.5		*p* = 0.67
	*N* = 27	*N* = 10			
Happiness	8.15 (2.67)	8.00 (2.45)	*U* = 147.00		*p* = 0.70
	*N* = 27	*N* = 10			
Hope	9.19 (1.59)	9.10 (1.28)	*U* = 148.5		*p* = 0.65
	*N* = 27	*N* = 10			
Calm	8.93 (1.44)	9.40 (0.84)	*U* = 118.5		*p* = 0.58
	*N* = 27	*N* = 10			
Interest in attending GC	27 (100%)	9 (90%)		*χ* ^2^ = 2.77	*p* = 0.09
	*N* = 27	*N* = 10			

* *p* < 0.05.

** *p* < 0.001.

#### 3.2.2 Reactions to the Video

There were statistically significant differences in the item “*The video made me feel distress”* between those who attended versus those who did not attend genetic counseling. Participants who did not attend counseling reported statistically significantly higher distress after watching the video (*M* = 4.60, SD = 3.53) compared to those who did attend (*M* = 2.26, SD = 2.34), (*U* = 73.5, *p* = 0.03). The other five emotions elicited by the video—*sadness*, *fear, happiness, calm, hopeful—*did not show significant differences between groups. Similarly, there were no statistically significant differences between groups in transportation (*U* = 139.5, *p =* 0.74) and identification with the main character (*U* = 124.5, *p* = 0.72).

#### 3.2.3 Logistic Regression

We employed a logistic regression to explore the independent association of distress elicited by the video with genetic counseling participation. The overall model significantly predicted genetic counseling participation (*χ* ^2^ = 4.61; *p =* 0.031), explaining 17.2% of the variation in the dependent variable (attended a pre‐test genetic counseling appointment) and correctly identified 81.1% of cases (40% of those who do not attend and 96.3% of those who do attend). Distress elicted by the video was significant predictor of genetic counseling uptake (*β* = −0.27, *p* = 0.037, CI 95% 0.58–0.98). The more distress that participants reported feeling after watching the video, the less likely they were to attend genetic counseling.

## 4 Discussion

In our study, 27 (72.9%) out of 37 at‐risk Latinas attended a genetic counseling session, and 25 (92.59%) of those completed genetic testing. One additional participant received genetic testing through her provider without receiving genetic counseling. While we are unaware of any currently available national estimates regarding the rate at which Latinx or Hispanic populations receive genetic counseling in the United States, this percentage is notably higher than previously reported interventions in similar populations [35, 36, 37, 38], and comparable only to one prior study evaluating culturally targeted genomic education tools with Latinx women in Miami and Puerto Rico [12]. Moreover, the 92.59% genetic testing uptake among those who attended counseling in our study is encouraging, given disparities in genetic testing uptake [1].

We found no statistically significant differences between women who attended counseling (*N* = 27) versus those who did not (*N* = 10) in sociodemographic variables, transportation, and identification with the main character. When looking at knowledge, there was a statistically significant difference in baseline knowledge measured by the GU‐Scale; more women who reported higher knowledge at baseline, went to counseling. However, this difference in baseline knowledge was not replicated by the Moffit scale measure. Importantly, all participants had an increase in knowledge [31], but there were no differences in change in knowledge between those who attended and did not attend counseling.

In our study, distress elicited by the video was predictive of genetic counseling uptake. Participants who reported feeling higher distress elicited by the video were less likely to attend genetic counseling at the 3 month follow up. These results are consistent with prior literature suggesting that narrative communication is a promising tool for behavior change in cancer care [19]. The pathways by which narratives result in behavior change are not well understood, but the role of constructs such as transportation, identification with characters, and emotions are the most commonly explored [18, 20, 34, 39]. Prior evidence indicates that narrative education can target *intention* and *emotion* better than didactic education can, as didactic education tends to target *knowledge*, *attitudes* and *beliefs* instead [16, 20]. Emotions are then potential mediators by which narrative communication could impact behavior change [20, 40].

Kreuter et al., 2007 propose that narrative communication can help by addressing emotional and existential issues. Individuals at increased risk for hereditary conditions are in a unique position because while they may not face a diagnosis personally, coping with their risk of cancer may elicit negative and ambivalent emotions [19]. Further, in a scoping review of narrative health communication, stories with stronger emotional appeal performed better than those with a didactic approach [16]. The theory of planned behavior, one of the most commonly cited foundational theories, posits that behavior is the result of intentions, attitudes, subjective norms, and perceived control [26]; yet, affective components, including emotions, are increasingly accepted as determinants of behavior beyond cognitive processes alone [16]. However, affective science research has been underutilized in cancer prevention and control [41].

In our video, emotions were intentionally addressed and integrated into the narrative story from the outset as detailed in Hurtado‐de Mendoza et al., 2020 [30]. Cancer risk information can increase distress in people with a higher genetic risk due to its personal relevance, but following up risk information that elicit negative emotions with messages that increase self‐efficacy can enhance behavioral change [42]. Thus, in the video, a message like *“you are at increased risk for HBOC”* is followed by *“you can meet with a genetic counselor.”*

Finally, the fact that our study utilized a video delivery may have also contributed to the high rate of genetic counseling participation. A 2015 meta‐analysis on the persuasive effects of narratives found that video narratives and those promoting prevention had more significant effects than print materials or messages promoting smoking cessation [43].

### 4.1 Limitations

Our study provides unique preliminary evidence for an under‐researched area crucial to addressing disparities with Spanish‐speaking Latina immigrants. However, it has some limitations. First, due to the design of the study, it is impossible to disentangle the effects of watching the narrative video plus the navigation component in their effects of GCT uptake, so no causal inferences about the intervention can me made.

Importantly, our study was conducted in close partnership with CBOs that had already established GCT referrals to free and low‐cost services for the Hispanic/Latinx population as part of their usual clinical care. Evidence suggests that patient navigation can help reduce systemic barriers in healthcare, increase screening and prevention strategies, and address unmet needs from historically underserved communities [44, 45]. Prior reports on the barriers and facilitators that Spanish‐speaking immigrant populations experience when accessing GCT in the US—including our formative research—emphasize both the role of both individual and systemic barriers [9, 27]. From a justice perspective, given the demographics of our sample and their specific needs, any individual‐level intervention may not be enough on its own to create meaningful and sustainable change to address health inequities; therefore, patient navigation was offered to all participants in our study. Future studies should be designed to address the confounding influence of narrative interventions plus patient navigation, evaluate Implementation outcomes, and assess costs.

Second, this pilot study had low statistical power due to its small sample size (*N* = 37). We need to be cautious in interpreting non‐significant associations as the lack of significance could be due to the fact that the study was not sufficiently powered to detect these differences. On the up side, the fact that we found statistically significant differences and a predictive variable despite the low power, supports their potential importance and warrant further review. Of note, the transportantion scale presented low internal validity. Future research should explore the use of different scales or validate adaptations for this population.

Third, participants were a small convenience sample from two partner CBOs in the Washington, DC, metropolitan area. Our high rate of genetic counseling uptake may be due to a self‐selection bias, given that enrolled women were already interested and willing to participate and may not generalize to Latinx in other regions of the US. Notably, of the 60 women initially scheduled for an in‐person appointment, only 40 showed up (see Figure 1). The self‐selection bias could be explained by other existing factors that motivated these 40 women to attend, especially as they were informed that they would be offered the opportunity to be navigated to free or low cost GCT services concluding the in‐person visit. Unfortunately, no data was collected from the 20 women who did not show to explore potential differences.

Fourth, the Latinx community is heterogeneous. While all participants in this study were born abroad, most participants were from Central America. This sample does not represent all Latinx lived experiences and lacks representation from Afro‐Latinx and individuals born in the Caribbean. Importantly, we did not ask about self‐identification as Indigenous. Future studies should plan to collect and report this information systematically. While this study had a single‐arm design, it is among the first to test the impact of culturally targeted educational interventions and patient navigation in GCT behavioral outcomes in this population. Future RCTs with larger samples, more diverse and representative sample of the Hispanic/Latinx population in different regions of the US and abroad are needed.

Lastly, reliance on self‐reported data and patient navigator records within a 3‐month timeframe may be subject to bias. Future studies should seek to confirm attendance to genetic counseling and completion of genetic testing through electronic medical records, directly with genetic counselors, or by reviewing laboratory results. Additionally, while assessing sustained outcomes beyond uptake of GCT was outside the scope of this study, future research should explore the potential impact of narrative education on other outcomes, such as cascade testing and adherence to surveillance recommendations.

### 4.2 Implications for Practice

Health communication efforts should consider leveraging narratives from the outset and recognizing the role of emotions in behavior. Importantly, emotions are influenced by culture [46, 47], and researchers must not assume they are familiar with all of them. In working with diverse populations, understanding and addressing perceptions of emotions in the cancer experience should be considered a priority. Additionally, due to the pervasive multi‐level barriers that the immigrant Hispanic population faces to receiving care, ensuring that the CBOs that serve them build capacity and integrate GCT navigation services into their clinical practice facilitates sustainability and optimizes the reach and impact of interventions.

## 5 Conclusion

Our study provides preliminary evidence that culturally targeted narrative videos along with patient navigation are a promising strategy to enhance GT uptake among Spanish‐speaking Latina immigrants. Future randomized controlled trials further exploring this approach are critical in order to move this field forward and ensure that advances in genomic medicine are made available to all.

## Author Contributions

**Sara Gómez‐Trillos:** investigation, project administration, data curation, writing original draft, review and editing. **Pilar Carrera** and **Amparo Caballero:** data curation, analysis, funding acquisition, reviewing and editing. **Vanessa B. Sheppard, Kristi D. Graves, Beth N. Peshkin,** and **Marc D. Schwartz:** conceptualization, methodology, funding acquisition, supervisión, review and editing. **Claudia Campos** and **Nathaly Garcés:** investigation, and resources. **Alejandra Hurtado de Mendoza:** conceptualization, methodology, funding acquisition, resourcing, supervisión, formal analysis, writing original draft, review and editing.

## Conflicts of Interest

The authors declare no conflicts of interest.

## References

[1] E. Chapman‐Davis , Z. N. Zhou , J. C. Fields , et al., “Racial and Ethnic Disparities in Genetic Testing at a Hereditary Breast and Ovarian Cancer Center,” Journal of General Internal Medicine 36, no. 1 (January 2021): 35–42, 10.1007/s11606-020-06064-x. 32720237 PMC7859010

[2] C. Zeng , L. A. Bastarache , R. Tao , et al., “Association of Pathogenic Variants in Hereditary Cancer Genes With Multiple Diseases,” JAMA Oncology 8, no. 6 (June 2022): 835–844, 10.1001/jamaoncol.2022.0373. 35446370 PMC9026237

[3] R. Yoshida , “Hereditary Breast and Ovarian Cancer (HBOC): Review of Its Molecular Characteristics, Screening, Treatment, and Prognosis,” Breast Cancer 28, no. 6 (November 2021): 1167–1180, 10.1007/s12282-020-01148-2. 32862296 PMC8514387

[4] E. Cabrera , I. Blanco , C. Yagüe , and A. Zabalegui , “The Impact of Genetic Counseling on Knowledge and Emotional Responses in Spanish Population With Family History of Breast Cancer,” Patient Education and Counseling 78, no. 3 (March 2010): 382–388, 10.1016/j.pec.2009.10.032. 19948386

[5] H. D. Nelson , M. Pappas , A. Cantor , E. Haney , and R. Holmes , “Risk Assessment, Genetic Counseling, and Genetic Testing for BRCA‐Related Cancer in Women: Updated Evidence Report and Systematic Review for the US Preventive Services Task Force,” JAMA 322, no. 7 (August 2019): 666–685, 10.1001/jama.2019.8430. 31429902

[6] D. Cragun , A. Weidner , C. Lewis , et al., “Racial Disparities in BRCA Testing and Cancer Risk Management Across a Population‐Based Sample of Young Breast Cancer Survivors,” Cancer 123, no. 13 (2017): 2497–2505, 10.1002/cncr.30621. 28182268 PMC5474124

[7] S. Reid , S. Cadiz , and T. Pal , “Disparities in Genetic Testing and Care Among Black Women With Hereditary Breast Cancer,” Current Breast Cancer Reports 12, no. 3 (September 2020): 125–131, 10.1007/s12609-020-00364-1. 33603954 PMC7885902

[8] D. E. Levy , S. D. Byfield , C. B. Comstock , et al., “Underutilization of BRCA1/2 Testing to Guide Breast Cancer Treatment: Black and Hispanic Women Particularly at Risk,” Genetics in Medicine 13, no. 4 (2011): 349–355, 10.1097/gim.0b013e3182091ba4. 21358336 PMC3604880

[9] H. A. Dron , D. Bucio , J. L. Young , H. K. Tabor , and M. K. Cho , “Latinx Attitudes, Barriers, and Experiences With Genetic Counseling and Testing: A Systematic Review,” Journal of Genetic Counseling 32, no. 1 (2023): 166–181, 10.1002/jgc4.1632. 36301246 PMC10091969

[10] K. M. Sussner , L. Jandorf , H. S. Thompson , and H. B. Valdimarsdottir , “Interest and Beliefs About BRCA Genetic Counseling Among At‐Risk Latinas in New York City,” Journal of Genetic Counseling 19, no. 3 (2010): 255–268, 10.1007/s10897-010-9282-4. 20151317 PMC4403243

[11] G. Joseph , M. S. Beattie , R. Lee , et al., “Pre‐counseling Education for Low Literacy Women at Risk of Hereditary Breast and Ovarian Cancer (HBOC): Patient Experiences Using the Cancer Risk Education Intervention Tool (CREdIT),” Journal of Genetic Counseling 19, no. 5 (October 2010): 447–462, 10.1007/s10897-010-9303-3. 20490636 PMC2944955

[12] C. C. Conley , E. M. Castro‐Figueroa , L. Moreno , et al., “A Pilot Randomized Trial of an Educational Intervention to Increase Genetic Counseling and Genetic Testing Among Latina Breast Cancer Survivors,” Journal of Genetic Counseling 30, no. 2 (2021): 394–405, 10.1002/jgc4.1324. 32936981 PMC7960565

[13] A. Hurtado‐de‐Mendoza , V. F. Reyna , C. R. Wolfe , et al., “Adapting a Theoretically‐Based Intervention for Underserved Clinical Populations at Increased Risk for Hereditary Cancer: Lessons Learned From the BRCA‐Gist Experience,” Preventive Medicine Reports 28 (August 2022): 101887, 10.1016/j.pmedr.2022.101887. 35855922 PMC9287635

[14] M. W. Kreuter , K. Steger‐May , S. Bobra , et al., “Sociocultural Characteristics and Responses to Cancer Education Materials Among African American Women,” Cancer Control 10, no. 5 (2003): 69–80, 10.1177/107327480301005s10. 14581907

[15] M. B. Moran , L. B. Frank , J. S. Chatterjee , S. T. Murphy , and L. Baezconde‐Garbanati , “A Pilot Test of the Acceptability and Efficacy of Narrative and Non‐Narrative Health Education Materials in a Low Health Literacy Population,” Journal of Communication in Healthcare 9, no. 1 (2016): 40–48, 10.1080/17538068.2015.1126995. 27872657 PMC5115781

[16] M. Z. Dudley , G. K. Squires , T. M. Petroske , S. Dawson , and J. Brewer , “The Use of Narrative in Science and Health Communication: A Scoping Review,” Patient Education and Counseling 112 (July 2023): 107752, 10.1016/j.pec.2023.107752. 37068426

[17] C. Funk and M. H. Lopez , A Brief Statistical Portrait of U.S. Hispanics. [Internet] (Pew Research Center, 2022): [cited 2024 Feb 22]. Available from:, https://www.pewresearch.org/science/2022/06/14/a‐brief‐statistical‐portrait‐of‐u‐s‐hispanics/.

[18] J. H. Yoo , M. W. Kreuter , C. Lai , and Q. Fu , “Understanding Narrative Effects: The Role of Discrete Negative Emotions on Message Processing and Attitudes Among Low‐Income African American Women,” Health Communication 29, no. 5 (2014): 494–504, 10.1080/10410236.2013.776001. 24111724 PMC4070308

[19] M. W. Kreuter , M. C. Green , J. N. Cappella , et al., “Narrative Communication in Cancer Prevention and Control: A Framework to Guide Research and Application,” Annals of Behavioral Medicine 33, no. 3 (September 2007): 221–235, 10.1007/bf02879904. 17600449

[20] S. T. Murphy , L. B. Frank , M. B. Moran , and P. Patnoe‐Woodley , “Involved, Transported, or Emotional? Exploring the Determinants of Change in Knowledge, Attitudes, and Behavior in Entertainment‐Education,” Journal of Communication 61, no. 3 (June 2011): 407–431, 10.1111/j.1460-2466.2011.01554.x.

[21] M. C. Green and T. C. Brock , “The Role of Transportation in the Persuasiveness of Public Narratives,” Journal of Personality and Social Psychology 79, no. 5 (November 2000): 701–721, 10.1037/0022-3514.79.5.701. 11079236

[22] K. G. Meilleur and M. T. Littleton‐Kearney , “Interventions to Improve Patient Education Regarding Multifactorial Genetic Conditions: A Systematic Review,” American Journal of Medical Genetics, Part A 149A, no. 4 (February 2009): 819–830, 10.1002/ajmg.a.32723. 19291763 PMC2776676

[23] T. S. Nolan , A. M. Bell , Y. N. Chan , A. L. Bryant , J. S. Bissram , and R. Hirschey , “Use of Video Education Interventions to Increase Racial and Ethnic Diversity in Cancer Clinical Trials: A Systematic Review,” Worldviews on Evidence‐Based Nursing 18, no. 5 (October 2021): 302–309, 10.1111/wvn.12539. 34561957 PMC8483572

[24] L. A. Baezconde‐Garbanati , J. S. Chatterjee , L. B. Frank , et al., “Tamale Lesson: A Case Study of a Narrative Health Communication Intervention,” Journal of Communication in Healthcare 7, no. 2 (July 2014): 82–92, 10.1179/1753807614y.0000000055.

[25] V. Henderson , J. M. Madrigal , L. C. Kendall , et al., “Pilot Study of a Culturally Sensitive Intervention to Promote Genetic Counseling for Breast Cancer Risk,” BMC Health Services Research 22, no. 1 (June 2022): 826, 10.1186/s12913-022-08193-x. 35752812 PMC9233847

[26] I. Ajzen , “The Theory of Planned Behavior,” Organizational Behavior and Human Decision Processes 50, no. 2 (December 1991): 179–211, 10.1016/0749-5978(91)90020-t.

[27] S. Gómez‐Trillos , V. B. Sheppard , K. D. Graves , et al., “Latinas’ Knowledge of and Experiences With Genetic Cancer Risk Assessment: Barriers and Facilitators,” Journal of Genetic Counseling 29, no. 4 (2020): 505–517, 10.1002/jgc4.1201. 31883202

[28] A. Hurtado‐de‐Mendoza , K. Graves , S. Gómez‐Trillos , et al., “Provider’s Perceptions of Barriers and Facilitators for Latinas to Participate in Genetic Cancer Risk Assessment for Hereditary Breast and Ovarian Cancer,” Healthcare 6, no. 3 (September 2018): 116, 10.3390/healthcare6030116. 30227649 PMC6164735

[29] A. Hurtado‐de‐Mendoza , S. Gómez‐Trillos , K. D. Graves , et al., “Process Evaluation of a Culturally Targeted Video for Latinas at Risk of Hereditary Breast and Ovarian Cancer,” Journal of Genetic Counseling 30, no. 3 (2021): 730–741, 10.1002/jgc4.1361. 33222313 PMC10226534

[30] A. Hurtado‐de‐Mendoza , K. D. Graves , S. Gómez‐Trillos , et al., “Developing a Culturally Targeted Video to Enhance the Use of Genetic Counseling in Latina Women at Increased Risk for Hereditary Breast and Ovarian Cancer,” Journal of Community Genetics 11, no. 1 (January 2020): 85–99, 10.1007/s12687-019-00423-w. 31104207 PMC6962403

[31] A. Hurtado‐de‐Mendoza , K. D. Graves , S. Gómez‐Trillos , et al., “Culturally Targeted Video Improves Psychosocial Outcomes in Latina Women at Risk of Hereditary Breast and Ovarian Cancer,” International Journal of Environmental Research and Public Health 16, no. 23 (January 2019): 4793, 10.3390/ijerph16234793. 31795362 PMC6926842

[32] P. Carrera , V. Sheppard , A. Caballero , S. Gómez‐Trillos , and A. Hurtado de Mendoza , “A Culturally Targeted Video to Promote Genetic Counseling in a Community Sample of At‐risk US Latina Women: The Role of the Concrete Mindset,” Journal of Community Psychology, [Internet] 50, no. 3 (2022): 1331–1342: [cited 2024 Feb 27]; Available from:, 10.1002/jcop.22718. 34606624

[33] M. L. Kasting , C. C. Conley , A. I. Hoogland , et al., “A Randomized Controlled Intervention to Promote Readiness to Genetic Counseling for Breast Cancer Survivors,” Psycho‐Oncology 28, no. 5 (2019): 980–988, 10.1002/pon.5059. 30883986 PMC6873464

[34] S. T. Murphy , L. B. Frank , J. S. Chatterjee , and L. Baezconde‐Garbanati , “Narrative Versus Non‐Narrative: The Role of Identification, Transportation and Emotion in Reducing Health Disparities,” Journal of Communication 63, no. 1 (February 2013): 116–137, 10.1111/jcom.12007. PMC3857102 24347679

[35] C. Dash , M. G. Mills , T. D. Jones , et al., “Design and Pilot Implementation of the Achieving Cancer Equity Through Identification, Testing, and Screening (ACE‐ITS) Program in an Urban Underresourced Population,” supplement, Cancer 129, no. S19 (2023): 3141–3151, 10.1002/cncr.34691. 37691526 PMC10502953

[36] D. Mays , M. E. Sharff , T. A. DeMarco , et al., “Outcomes of a Systems‐Level Intervention Offering Breast Cancer Risk Assessments to Low‐Income Underserved Women,” Familial Cancer 11, no. 3 (2012): 493–502, 10.1007/s10689-012-9541-7. 22711611 PMC3521596

[37] R. Kukafka , S. Pan , T. Silverman , et al., “Patient and Clinician Decision Support to Increase Genetic Counseling for Hereditary Breast and Ovarian Cancer Syndrome in Primary Care: A Cluster Randomized Clinical Trial,” JAMA Network Open 5, no. 7 (July 2022): e2222092, 10.1001/jamanetworkopen.2022.22092. 35849397 PMC9294997

[38] J. An , J. McDougall , Y. Lin , et al., “Randomized Trial Promoting Cancer Genetic Risk Assessment When Genetic Counseling Cost Removed: 1‐Year Follow‐Up,” JNCI Cancer Spectrum 8, no. 2 (April 2024): pkae018, 10.1093/jncics/pkae018. 38490263 PMC11006111

[39] L. A. Baezconde‐Garbanati , J. S. Chatterjee , L. B. Frank , et al., “ *Tamale Lesson* : A Case Study of a Narrative Health Communication Intervention,” Journal of Communication in Healthcare 7, no. 2 (July 2014): 82–92, 10.1179/1753807614y.0000000055.

[40] A. J. Dillard , R. A. Ferrer , and J. D. Welch , “Associations Between Narrative Transportation, Risk Perception and Behaviour Intentions Following Narrative Messages About Skin Cancer,” Psychology and Health 33, no. 5 (2018): 573–593, 10.1080/08870446.2017.1380811. 28975805

[41] R. A. Ferrer , P. A. Green , and L. F. Barrett , “Affective Science Perspectives on Cancer Control: Strategically Crafting a Mutually Beneficial Research Agenda,” Perspectives on Psychological Science 10, no. 3 (May 2015): 328–345, 10.1177/1745691615576755. 25987511 PMC4438787

[42] K. Witte and M. Allen , “A Meta‐Analysis of Fear Appeals: Implications for Effective Public Health Campaigns,” Health Education & Behavior 27, no. 5 (October 2000): 591–615, 10.1177/109019810002700506. 11009129

[43] F. Shen , V. C. Sheer , and R. Li , “Impact of Narratives on Persuasion in Health Communication: A Meta‐Analysis,” Journal of Advertising 44, no. 2 (April 2015): 105–113, 10.1080/00913367.2015.1018467.

[44] Jang J. , K. H. Sun , K. Mann , et al., “Patient Navigation Increases Breast, Cervical, and Colorectal Cancer Screening Among Immigrants in the U.S.: A Systematic Review,” Journal of General Internal Medicine [Internet], 40, no. 10 (May 2025): 2358–2368: [cited 2025 July 20]; Available from:, 10.1007/s11606-025-09566-8. 40329031 PMC12344048

[45] R. J. Chan , V. E. Milch , F. Crawford‐Williams , et al., “Patient Navigation Across the Cancer Care Continuum: An Overview of Systematic Reviews and Emerging Literature,” CA: A Cancer Journal for Clinicians 73, no. 6 (2023): 565–589, 10.3322/caac.21788. 37358040

[46] Kitayama S. , Markus H. R. , eds., Emotion and Culture: Empirical Studies of Mutual Influence (American Psychological Association, 1994), 385.

[47] H. R. Markus and S. Kitayama , “The Cultural Shaping of Emotion: A Conceptual Framework,” in Emotion and Culture: Empirical Studies of Mutual Influence (American Psychological Association, 1994), 339–351.

